# Optimizing Treatment of Familial Hypercholesterolemia in Children and Adolescents

**DOI:** 10.1007/s11886-015-0629-1

**Published:** 2015-08-15

**Authors:** Ilse K. Luirink, Barbara A. Hutten, Albert Wiegman

**Affiliations:** Department of Pediatrics, Emma Children’s Hospital, Academic Medical Center, University of Amsterdam, Meibergdreef 9, 1105 AZ Amsterdam, The Netherlands; Department of Clinical Epidemiology, Biostatistics and Bioinformatics, Academic Medical Center, University of Amsterdam, Meibergdreef 9, 1105 AZ Amsterdam, The Netherlands; Department of Vascular Medicine, Academic Medical Center, University of Amsterdam, Meibergdreef 9, 1105 AZ Amsterdam, The Netherlands

**Keywords:** Familial hypercholesterolemia, Children, Adolescents, LDL cholesterol, Treatment, Novel agents

## Abstract

Cardiovascular disease (CVD) is still the most prominent cause of death and morbidity in the world, and one of the major risk factors for developing CVD is hypercholesterolemia. Familial hypercholesterolemia (FH) is a dominantly inherited disorder characterized by markedly elevated plasma low-density lipoprotein cholesterol and premature coronary heart disease. Currently, several treatment options are available for children with FH. Lifestyle adjustments are the first step in treatment. If this is not sufficient, statins are the preferred initial pharmacological therapy and they have been proven effective and safe. However, treatment goals are often not achieved and, hence, there is a need for novel treatment options. Currently, several options are being studied in adults and first results are promising. However, studies in children are still to be awaited.

## Introduction

Familial hypercholesterolemia (FH) is a common dominantly inherited disorder of lipoprotein metabolism. It is caused by mutations in genes encoding key proteins involved in the low-density lipoprotein receptor (LDLR) and its endocytic and recycling pathways. In the vast majority (>90 %), FH is due to a loss of function mutations in the LDLR gene. In addition, FH can be caused by mutations in the apolipoprotein B (ApoB) (5 %) and proprotein convertase subtilisin-like kexin type 9 (PCSK9) (1 %) genes [1–3]. The associated decrease in the function of LDLR results in a decreased rate of low-density lipoprotein cholesterol (LDL-C) removal from the circulation and, thus, in an increase of plasma LDL-C.

Historically, the prevalence is estimated at 1:500 for clinical heterozygous FH (HeFH) and at 1:1.000.000 for homozygous FH (HoFH). However, recent studies suggest that the actual prevalence is higher—approximately 1:250 for HeFH and 1:300.000 for HoFH [4••, 5•, 6••]. Even higher prevalence is observed in subpopulations such as Afrikaners in South Africa, French Canadians, or Christian Lebanese due to founder effects [7].

Diagnosis in children should preferably be established by detection of the FH-causing mutation, which is considered to be the gold standard for diagnosis. Genetic testing, however, is not always available. In that case, FH in children can be diagnosed phenotypically by the presence of an increased LDL-C level plus a family history of premature coronary heart disease (CHD) or elevated LDL-C levels compatible with FH (Table 1).

**Table 1:** Diagnosis of familial hypercholesterolemia in children and adolescents

- Family history of premature CHD plus high LDL-C levels are the two key selective screening criteria^a^
- Cholesterol testing should be used to make a phenotypic diagnosis
- An LDL-C level ≥5 mmol/L (190 mg/dL) on two occasions after a 3-month diet indicates a high probability of FH. A family history of premature coronary heart disease in close relative(s) and/or baseline high cholesterol in one parent, together with an LDL-C level ≥4 mmol/L (160 mg/dL), indicates a high probability of FH. If the parent has a genetic diagnosis, an LDL-C level ≥3.5 mmol/L (130 mg/dL) suggests FH in the child
- Secondary causes of hypercholesterolemia should be ruled out
- DNA testing establishes the diagnosis. If a pathogenic LDLR mutation is identified in a first-degree relative, children may also be genetically tested
- If a parent died from CHD, a child even with moderate hypercholesterolemia should be tested genetically for FH and inherited elevation of Lp(a)

^a^Acknowledgement to the FH Foundation (http:/thefhfoundation.org/) (with permission from [26••])

Currently, there are three strategies to consider regarding detection of FH in children: cascade screening, universal screening, or selective screening based on family history [8–10]. Different countries use different strategies, based on feasibility and opinions of local expert groups [11••]. For example, in Australia and in most European countries, cascade screening based on genetic testing is recommended, whereas in the USA, selective screening beginning at the age of 2 years and universal screening at the age of 9–11 years are advocated [9, 11••, 12, 13].

In this paper, we focus on FH. However, atherosclerosis and its clinical consequence are multifactorial in origin. Therefore, it is important in patients with FH to pay sufficient attention to all the other risk factors that constitute their total CV risk. In children and adolescents, it is essential to abstain from tobacco smoking, to exercise regularly, and to keep up with a well-balanced and healthy diet, for the primordial and primary prevention of cardiovascular disease (CVD).

This review aims to provide an overview of the current treatment options for pediatric patients with FH plus emerging novel agents.

## FH and Cardiovascular Risk

CVD is the leading cause of death and morbidity worldwide, and an important risk factor for its development is the presence of hypercholesterolemia.

Severely elevated LDL-C levels from birth onwards accelerate the development of atherosclerotic CVD, especially CHD. Although cardiovascular events are rare in childhood, children with FH already show functional and morphological changes of the vessel wall [11••, 14]. Carotid intima-media thickness (cIMT) and flow-mediated dilatation (FMD), both surrogate markers for atherosclerosis, are respectively increased and impaired in children with FH compared to healthy controls [14, 15]. The difference in mean cIMT between children with FH and unaffected siblings may even be significant as early as the age of 8 years as recent research shows [16•].

These findings indicate that early treatment of FH is necessary to reduce the risk of CHD later in life.

## Non-pharmacological Treatment

Lifestyle interventions that focus on lowering cholesterol and reducing other known cardiovascular risk factors are the first step in treating children with FH. Important interventions are dietary change, exercise, and cessation of smoking.

The latest cardiovascular health guidelines for pediatric patients by the National Heart, Lung and Blood Institute (NHLBI) recommend the CHILD-2 diet for children with known hypercholesterolemia [12]. This is a fat-modified diet, which limits the saturated fat intake to 7 % of total daily calories and cholesterol intake too less than 200 mg/day. This has been shown to be safe and modestly effective in lowering LDL-C level [12, 17, 18].

Dietary supplementation with plant sterols or stanols might enhance LDL-C-lowering effects. However, long-term studies on effect and safety have not been completed and most studies done in children are small in size [12, 19]. Furthermore, a recent systematic review about dietary interventions in children and adults with FH stated that no conclusions can be made about the effectiveness of a cholesterol-lowering diet or any of the other dietary interventions suggested for FH on the prevention of CVD [20]. Currently, the use of these substances is only recommended for children from the age of 5 years [21].

Physical activity in children with FH should be promoted. Although data on the effect in children is limited, increasing physical activity might improve fasting lipid profiles [12, 22].

Strong discouragement of smoking is important, as smoking is strongly associated with an increased risk of CVD, especially in combination with hypercholesterolemia [11••, 23].

## Pharmacological Treatment

Lifestyle interventions alone are rarely sufficient in children with FH. Pharmacological treatment may be considered when cholesterol levels are not significantly reduced after 3 to 6 months of leading a suitable lifestyle.

Good evidence for an absolute or relative target does not exist in children, nor in adults. Current expert opinion recommends plasma LDL-C targets for children aged 8–10 years to be <4 mmol/L and for children older than 10 years to be <3.5 mmol/L [9, 11••, 13, 24, 25]. In a recent consensus paper on FH in children, an ideal reduction of 50 % from pre-treatment levels for children aged 8 to 10 years has been recommended [26••].

### Statins

All statins competitively inhibit the enzyme HMG-CoA reductase. This is a rate-limiting enzyme in the metabolic pathway of cholesterol biosynthesis in the liver. By inhibiting this step, there is a reduction of intracellular cholesterol, which, in turn, leads to an upregulation of LDL receptors on the cell surface of hepatocytes, thus increasing LDL-C clearance from the circulation [27].

Current expert consensus recommends the initiation of statin therapy in HeFH between 8 and 10 years. Pravastatin is approved for children with FH aged from 8 years [4••, 11••]. At this moment, FDA and European Medicines Agency (EMA) approval exist for simvastatin, lovastatin, atorvastatin, fluvastatin, and rosuvastatin from 10 years onwards. A recently published article shows that pitavastatin is effective and safe in children between 6 and 17 years [28]. When LDL-C levels are very high or there are additional cardiovascular risk factors, treatment needs to be initiated early [29]. Several systematic reviews and meta-analyses of statin therapy in children with FH have shown statins to be effective with LDL-C reductions between 21 and 39 % depending on the dose and the type of statin used. In addition, they are considered safe with no significant differences in adverse events, growth, or sexual development between the statin-treated and placebo-treated children [30–32]. Furthermore, cIMT and vascular endothelial function, both markers of early atherosclerosis, improve when children with FH receive statin therapy [14, 15].

Adverse events are not frequent in children but can include muscle cramps, GI complaints, an increase in liver transaminase levels and, very rarely, rhabdomyolysis.

Statin treatment should be initiated at the lowest recommended dose and should only be started after two separate LDL-C measurements. It is recommended to up-titrate according to the LDL-C-lowering effect and tolerability [11••, 24, 25].

Prior to initiating statin therapy, liver transaminases, CK, and a baseline fasting lipid panel should be measured. After starting treatment, growth, weight, and physical and sexual development should be monitored [11••]. Furthermore, parents and child should be well informed about the possible side effects, so they can report any drug-related complaints in time [12]. Clinicians should be aware of drug interactions, especially with cyclosporine, erythromycin, and gemfibrozil.

When contemplating or at risk of pregnancy, adolescent girls should be counseled to stop statin treatment based on their potential teratogenic effects. A recent systematic review suggested that statins are unlikely to be teratogenic. However, large trials are lacking and the current consensus is to stop statins during pregnancy [33, 34].

All in all, statins are still the cornerstone of FH treatment and they appear to be safe and effective in children. However, most studies done in children had a short-term follow-up period. Recently, a study on statins with 10 years of follow-up has been published, the longest follow-up study thus far [35••]. This showed that long-term statin treatment initiated in childhood was associated with normalization of cIMT progression during aging. In terms of long-term safety, this study did not reveal a significant difference in laboratory parameters and overall statin therapy was well tolerated.

### Ezetimibe

Ezetimibe is a selective cholesterol absorption inhibitor that acts at the brush border of the small intestine and inhibits the uptake of dietary and biliary cholesterol into the enterocytes. This reduces the delivery of cholesterol to the liver, which, by positive feedback, induces a compensatory increase in LDLRs, thus increasing the clearance of LDL-C from the circulation [36–38].

In adults, ezetimibe is most frequently used in combination with a statin where it provides an additional reduction of LDL-C of approximately 17 % and appears to be safe and well tolerated [39, 40].

A small number of short-term studies have looked at the efficacy and safety of ezetimibe in children. Both co-administered with a statin or as monotherapy, ezetimibe appears to effectively lower LDL-C without any significant side effects [41–44]. However, additional studies concerning long-term safety and efficacy are needed.

Ezetimibe 10 mg is registered by the FDA and the EMA for pediatric use from the age of 10 years onward. Currently, the main use in children and adolescents is for those patients who either fail to achieve target LDL-C levels on a statin alone or those who are unable to tolerate (high-dose) statins due to side effects [44].

### Bile Acid Sequestrants

Bile acid sequestrants (BAS) reduce plasma LDL-C by binding bile acids in the intestine and thus removing them from the enterohepatic cycle. As a result, there is an increase in the formation of bile acids from intrahepatic cholesterol. Consequently, the intracellular cholesterol concentration is reduced, which triggers the upregulation of LDLR on the surface of the hepatocyte.

They can lower LDL-C by 10–20 %, but, owing to frequent side effects (abdominal pain, nausea, and constipation) of the classical BAS, tolerability and long-term compliance are poor in pediatric patients. These agents also interfere with the absorption of folate and fat-soluble vitamins, and appropriate dietary supplementation is recommended in children [45].

A few years ago, a second-generation BAS, colesevelam, was evaluated in children with FH [46, 47]. Colesevelam has a greater affinity for bile salts and can thus be used in lower dosage. Children experience fewer side effects, which makes the long-term adherence better.

Currently, colesevelam is the only BAS approved for the treatment of pediatric patients with HeFH. Colesevelam comes in tablets and in powder form and can be used at dosages of 3.75 once or 1.875 g twice a day [48].

## Novel Agents

Even though there are currently different pharmacological therapeutic options in children, target values are often difficult to achieve and maintain. Findings of a recent study in the Netherlands show that only 21 % of the 1249 participating adult HeFH patients reached the target LDL-C levels, mainly because they were not treated with the highest dosages of statins, they are statin intolerant, or their levels were simply too high to be controlled with the current available therapy [49]. This stresses the urge for novel treatment options for patients with HeFH.

**Fig. 1:**
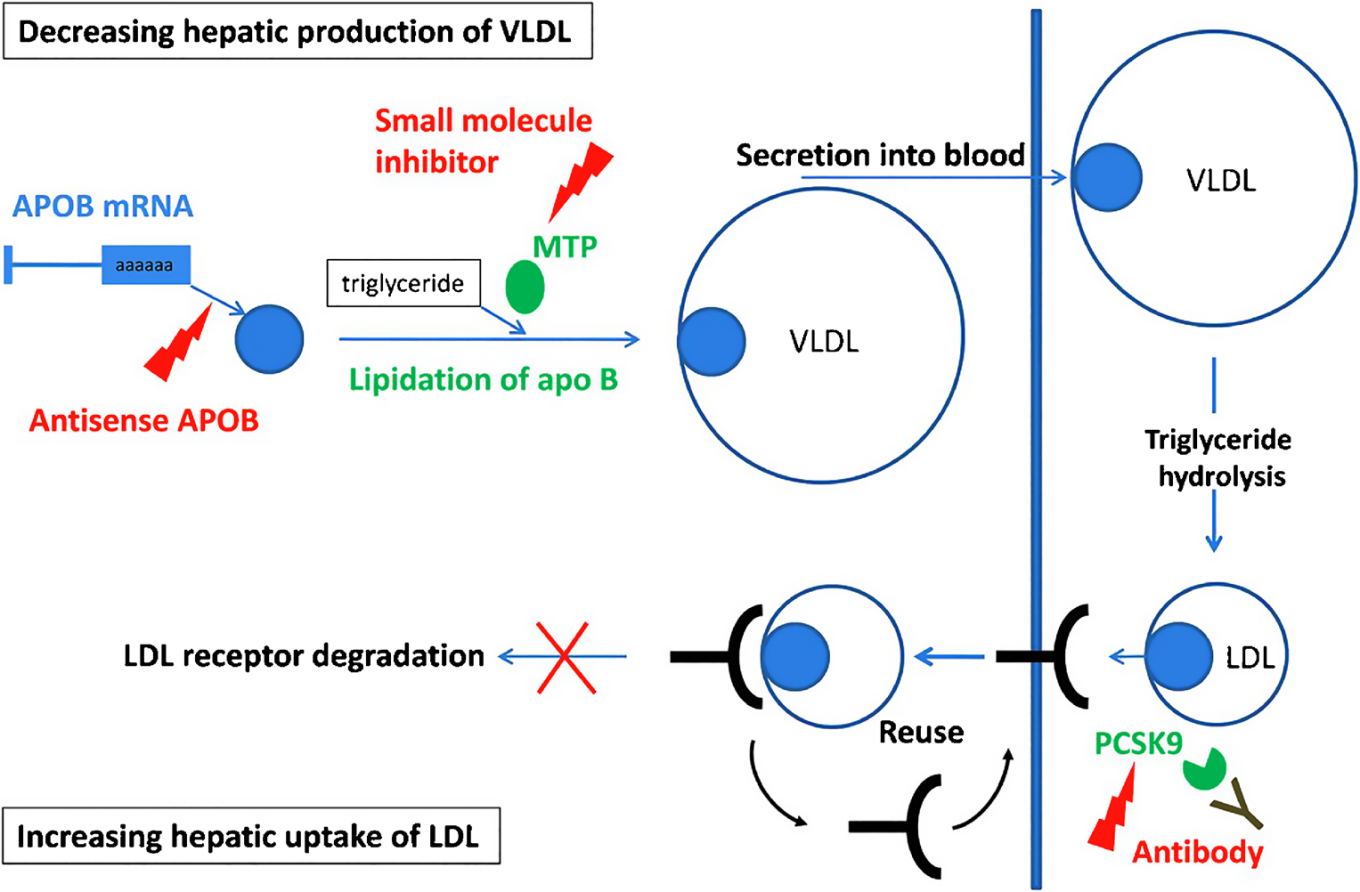
Novel lipid-regulating drug targets. Novel drugs target either very low-density lipoprotein (*VLDL*) production, by inhibiting apolipoprotein B synthesis [apolipoprotein B (*ApoB*) antisense oligonucleotide, mipomersen] or lipid loading onto nascent ApoB [microsomal triglyceride transfer protein (MTP) inhibitor, lomitapide], or low-density lipoprotein catabolism by increasing low-density lipoprotein receptor recycling (PCSK9 inhibitors) (with permission [6••])

Much is expected from the agents targeting PCSK9, antisense oligonucleotides targeting apolipoprotein B, and microsomal triglyceride transfer protein inhibitors to reduce LDL-C beyond the levels attainable with statin monotherapy [13] (Fig. 1). The safety and efficacy results of trials with these drugs in children are still awaited [29].

### PCSK9 Inhibitors

PCSK9 is a protease secreted by hepatocytes that regulates the LDLR [50, 51]. PCSK9 binds the LDLR and promotes its internalization/degradation in the hepatocyte. PCSK9 either prevents the recycling of the LDLR to the hepatocyte cell surface or it chaperones the LDLR to the lysosome where it is degraded [11••, 50].

In this way, PCSK9 acts as the terminator of the long life cycle of the LDLR, which spans hundreds of recycling events [51]. Gain-of-function mutations in PCSK9 lead to a phenotype typical for FH; conversely, dominant loss-of-function mutations result in an enhancement of LDLR activity and thus to lifelong reductions in plasma LDL-C and reduced risk of CHD [11••, 50–52]. This insight led to the rationale that inhibiting PCSK9 could be a therapeutic option for patients with FH.

Monoclonal antibodies against PCSK9 are currently being studied as therapeutic agents for FH patients. These antibodies prevent PCSK from productively interacting with the LDLR on the surface of hepatocytes, thus preventing its degradation [50].

Recently, two large phase III trials have shown that evolocumab and alirocumab can lower LDL-C by, respectively, an additional 61–62 % in patients with HeFH already treated with statins with or without other approved lipid-modifying therapies [53•, 54•]. Both studies performed a post hoc analysis that showed evidence of a reduction in cardiovascular events [53•, 54•]. In a study performed in statin-intolerant patients, evolocumab performed better with regard to lowering LDL-C levels when compared to ezetimibe [55]. All monoclonal antibodies are not yet approved for general use, and only one trial has been conducted that also included HoFH children so far [56]. Long-term efficacy and safety of these agents are yet to be investigated. However, clearly, these are very promising agents in the treatment of HeFH either as a monotherapy or as an adjuvant therapy [11••].

### Lomitapide

A second novel agent is lomitapide, an oral inhibitor of the microsomal triglyceride transport protein (MTP). MTP plays an important role in the formation of ApoB-containing lipoproteins in hepatocytes and enterocytes. Inhibiting MTP therefore leads to a reduced secretion of very low-density lipoprotein (VLDL) and chylomicrons, respectively, into the circulation [6••]. Lomitapide has demonstrated to reduce LDL-C levels by 50 % after 26 weeks of use in an open-label trial in HoFH patients already treated with lipid-lowering therapy [57]. Gastrointestinal symptoms and liver fat accumulation are the most frequently observed side effects [57, 58]. Currently, lomitapide is only licensed for the treatment of homozygous patients as an add-on therapy by both the FDA and the EMA. In children, lomitapide has yet to be studied.

### Mipomersen

Mipomersen is a second-generation antisense oligonucleotide that targets the mRNA of ApoB, thereby inhibiting its ribosomal translation [6••, 59]. ApoB is the main protein of LDL and its precursor VLDL; by reducing the synthesis of ApoB, mipomersen reduces the hepatic production and secretion of VLDL [6••]. In a placebo-controlled double-blind trial in HoFH patients, mipomersen resulted in a 25 % reduction in LDL-C from baseline [60]. In severe HeFH with coronary artery disease, mipomersen decreased LDL-C with 28 % [61]. In addition to frequent injection side reactions, fatigue and myalgia, mipomersen can induce hepatic steatosis [6••, 11••, 60, 61]. This agent has not been tested in children and has only been approved by the FDA, not the EMA, to be used in HoFH patients as an orphan drug.

## Homozygous FH

HoFH is clinically characterized by plasma LDL-C levels >13 mmol/L (>500 mg/dL), extensive xanthomatas, and marked premature and progressive atherosclerotic CVD [6••]. If not treated, most patients with these LDL-C levels will develop atherosclerosis before the age of 20 years and, most often, will not survive beyond the age of 30 [1].

True genetic homozygotes have identical mutations in both alleles of the affected gene, but most patients are compound heterozygotes with two different mutations in the LDLR, the ApoB or the PCSK9 gene [5•, 6••].

The severity of the clinical phenotype per patient depends on the residual LDLR activity. HoFH patients are classified as either receptor negative (<2 % residual activity) or receptor defective (2–25 % residual activity) [1, 62]. Residual activity is associated with the severity of disease; receptor-negative patients have higher LDL-C levels and poorer clinical prognosis [6••, 63]. Because of the aggressive nature of this disease, children who are suspected of HoFH should be referred to specialized centers as soon as possible.

To prevent or delay CVD in children with HoFH, early and aggressive cholesterol-lowering treatment is warranted [6••]. Starting with a statin, with or without ezetimibe, as soon as the diagnosis is clear delays cardiovascular events and prolongs survival as shown in a retrospective cohort study [64]. Lipoprotein apheresis, if available, is an important adjunctive treatment for HoFH and can improve CHD outcome [65•]. Lipoprotein apheresis refers to all extracorporeal methods used to remove atherogenic ApoB-containing lipoproteins from the circulation [66]. A single treatment can decrease plasma LDL-C by 55–70 % relative to pre-treatment levels [6••]. This reduction, however, is temporary, and treatment once per 1 or 2 weeks is therefore desirable [67, 68]. When performed once a week, mean LDL-C levels can come close or under target levels [6••]. Data on LDL apheresis in children is limited, but several case series and case reports show LDL apheresis to be a safe and effective treatment for HoFH in children [69–71]. Current guidelines recommend starting apheresis in those who need it as soon as possible, ideally by age 5, but no later than age 8 [6••].

As mentioned earlier, lomitapide and mipomersen are recently approved by the FDA to be used as an adjunct therapy for HoFH in patients aged ≥18 and ≥12 years, respectively. With both agents, fat accumulation in the liver has been observed and the long-term consequences of the use of these agents remain unclear. Therefore, the results of long-term clinical trials need to be awaited [72].

Antibody therapies to PCSK9 could be another future option for children with HoFH. In a pilot study performed in patients with HoFH, it was shown that in receptor-defective patients, a decrease of LDL-C of 26.3 % could be achieved. However, in the receptor-negative patients, no effect was seen [73]. Pediatric trials of monoclonal antibodies to PCSK9 are planned.

## Conclusion

Early identification and treatment of children with FH are important to prevent atherosclerosis at the earliest stage of development. Screening strategies differ across countries and depend on feasibility and local guidelines. Treatment of FH should start at a young age with lifestyle adjustments. If lifestyle changes alone are not sufficient, lipid-lowering treatment from the age of 8 is indicated, with statins as the first choice of treatment. In different short-term studies and one long-term follow-up study, statins are proven to be safe and effective. Safety, however, remains a really important issue, and more long-term follow-up studies in large cohorts are strongly recommended.

Current treatment options are not always sufficient, and new promising agents like PCSK9 inhibitors are currently being studied in phase III trials in adults. Trials with these novel agents in children are still awaited.
